# The Effects of Curcumin on Vascular Endothelial Function, Lipid Metabolism, Inflammation and Neuroprotection—A Review

**DOI:** 10.3390/nu18071032

**Published:** 2026-03-25

**Authors:** Mateusz Ozorowski, Michał Wiciński, Grzegorz Liczner, Jakub Wójcicki, Elżbieta Włodarczyk

**Affiliations:** 1 Department of Pharmacology and Therapeutics, Faculty of Medicine, Collegium Medicum in Bydgoszcz, Nicolaus Copernicus University, M. Curie 9, 85-090 Bydgoszcz, Poland; michal.wicinski@cm.umk.pl (M.W.); grzelicz@cm.umk.pl (G.L.); jakub.wojcicki@cm.umk.pl (J.W.); 2 Medical Faculty, Politechnika Bydgoska im. Jana i Jędrzeja Śniadeckich, Al. prof. S. Kaliskiego 7, 85-796 Bydgoszcz, Poland; ewlodar@yahoo.com

**Keywords:** curcumin, vascular endothelium, neuroprotection, lipids metabolism, inflammation, sepsis

## Abstract

Curcumin, a polyphenolic compound obtained from the rhizome of *Curcuma longa*, is the main bioactive component of turmeric and exhibits a wide range of biological properties. This naturally occurring polyphenolic compound is widely known for its protective properties on the vascular endothelium and its anti-inflammatory effects. Curcumin has been recognized as a factor in improving antioxidant defenses and lipid metabolism and as a neuroprotective agent. Thanks to its broad spectrum of activity, curcumin is gaining popularity as an ingredient in dietary supplements and as part of a healthy diet that supports overall health. In this article, we will take a closer look at curcumin—based on a review of the current literature, we will learn what curcumin is and what health benefits it can provide.

## 1. Introduction

Curcumin, a potent antioxidant and anti-inflammatory compound from the polyphenol group, may play a significant role in modulating endothelial function and the inflammatory processes that underlie many chronic diseases, such as cardiovascular and neurodegenerative diseases. *Curcuma longa* is grown in the southern and southwestern regions of Asia. It occupies an important place in the cuisine of Iran, Malaysia, India, China, Polynesia, and Thailand. It is used as a spice, and it affects the nature, color, and taste of food. Curry is the best-known spice that contains turmeric rhizome powder. Curcumin is also used as an ecological dye; it is known as Natural Yellow 3 and has been assigned the E number E100 when used as a food coloring agent. Numerous studies support its modulation of cellular and molecular processes associated with inflammatory responses and protection against oxidative damage. Atherosclerosis remains a leading cause of mortality worldwide due to conditions such as coronary heart disease, stroke, and peripheral artery disease. It is characterized by the accumulation of lipids, inflammatory cells, and fibrotic elements in arterial walls, leading to the formation of atherosclerotic plaques [1,2]. These plaques can impair arterial integrity and function, leading to reduced blood flow and potential plaque rupture, which can lead to acute cardiovascular events. In recent years, there has been growing interest in dietary curcumin as a therapeutic agent in the prevention and treatment of atherosclerosis [1,2,3]. This intensely yellow flavonoid, commonly found in the dried and ground rhizome of *Curcuma longa*, has gained significant attention due to its potent protective properties against vascular endothelial damage, metabolic disorders, neuroprotection, and chemokine-induced inflammation [4,5]. Curcumin has attracted considerable interest due to its diverse biological activities and potential therapeutic applications [6,7]. Curcumin modulates numerous intracellular signaling pathways, such as NF-κB, Nrf2, PI3K/Akt, and MAPK, thereby influencing inflammatory responses, cellular redox balance, and cell survival [1,2]. Accumulating evidence indicates that one of the key biological targets of curcumin is the vascular endothelium—a dynamic organ responsible for regulating vascular tone, hemostasis, inflammation, and angiogenesis. Under conditions of inflammation and oxidative stress, endothelial activation leads to increased expression of adhesion molecules and increased leukocyte recruitment to the vascular wall, contributing to vascular dysfunction. Curcumin inhibits NF-κB activation and attenuates oxidative stress in endothelial cells, resulting in reduced expression of pro-inflammatory cytokines and adhesion molecules and maintaining endothelial barrier integrity [3,5]. The endothelium plays a crucial role in maintaining vascular homeostasis through the balanced release of vasoactive mediators, including nitric oxide (NO), prostacyclin, and endothelin-1 [3]. Endothelial dysfunction is an early marker of atherosclerosis and other cardiovascular diseases, and the main underlying mechanisms are oxidative stress and chronic low-grade inflammation [4].

## 2. Materials and Methods

**Figure 1:**
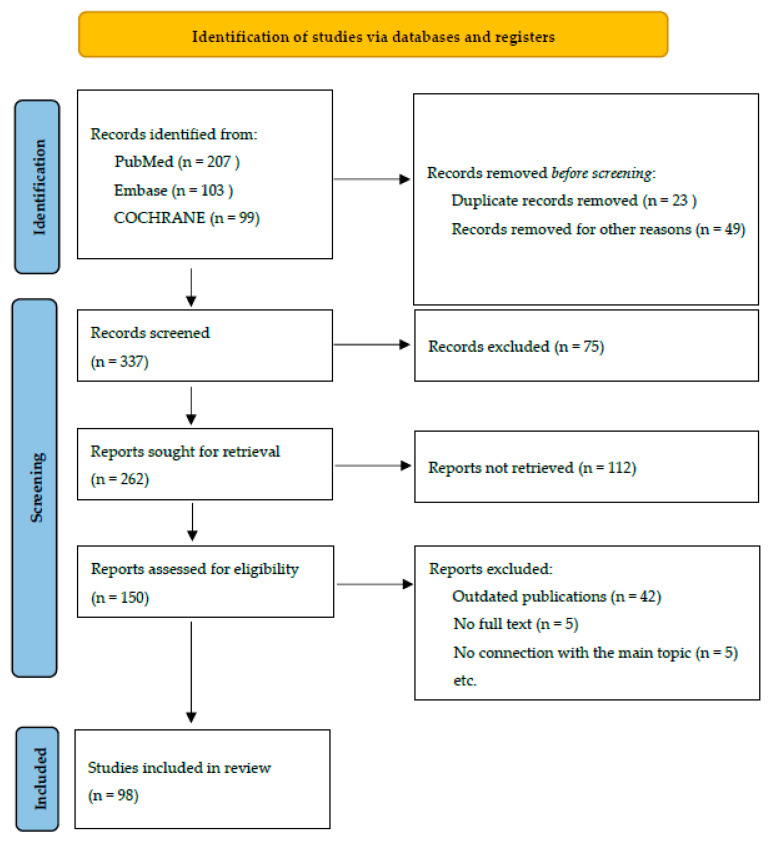
Study selection diagram (PRISMA).

This systematic review included recent clinical trials published in the PubMed, Embase, and COCHRANE databases. The keywords used were: *Curcuma longa*, curcumin, inflammation, lipid metabolism, SEPSIS, vascular endothelium, and neuroprotection. These keywords were used to identify studies related to curcumin. The search period was until 15 January 2026. Studies published before 2009 were excluded. The inclusion criteria included interventional studies involving humans and animals. The exclusion criteria included studies not published in English, editorials, conference papers, poster presentations, and case reports. Identification of studies using databases and registries was performed according to PRISMA guidelines and presented in Figure 1.

## 3. Vascular Endothelium

One of the most well-documented protective actions of curcumin on the endothelium is its ability to attenuate oxidative stress, as demonstrated in experimental models of endothelial injury and conditions associated with increased oxidative burden [5,8,9]. Oxidative stress is a central mechanism underlying endothelial dysfunction and is characterized by excessive production of reactive oxygen species (ROS) and impaired antioxidant defense. In endothelial cells, increased ROS generation leads to reduced nitric oxide (NO) bioavailability, uncoupling of endothelial nitric oxide synthase (eNOS), lipid peroxidation, and activation of redox-sensitive inflammatory pathways. Experimental studies have demonstrated that curcumin directly scavenges reactive oxygen species and upregulates endogenous antioxidant enzymes, including superoxide dismutase, catalase, and glutathione peroxidase, thereby restoring redox balance within the endothelium [5,8]. In models of metabolic and vascular injury, curcumin supplementation significantly reduced superoxide production, improved endothelial-dependent vasodilation, and attenuated structural and functional markers of endothelial damage [8]. Beyond its direct antioxidant effects, curcumin also modulates redox-sensitive signaling pathways involved in endothelial survival and stress responses, contributing to improved resistance of endothelial cells to ischemia–reperfusion injury and other cytotoxic insults [9]. Collectively, these findings indicate that attenuation of oxidative stress represents a key mechanism by which curcumin preserves endothelial integrity and function.

These observations are supported by a growing body of clinical evidence. Endothelial dysfunction is a key contributor to the pathogenesis of cardiovascular diseases such as atherosclerosis, hypertension, and coronary artery disease. Clinical studies indicate that curcumin supplementation improves endothelial function in both resistance and conduit vessels, primarily through enhanced NO bioavailability and reduced oxidative stress. Aging is associated with a progressive decline in endothelial function, even in the absence of overt cardiovascular disease. Available data suggest that curcumin supplementation may partially reverse age-related endothelial dysfunction by mitigating oxidative stress and restoring NO signaling pathways, supporting its potential role as a nutraceutical for vascular health in older adults [6,7]. These findings are further supported by recent evidence. An umbrella meta-analysis of randomized clinical trials demonstrated that curcumin supplementation is associated with significant improvements in biomarkers of endothelial function, oxidative stress, and systemic inflammation, although substantial heterogeneity among study designs, doses, and formulations was noted [10]. Key experimental and clinical studies assessing the effects of curcumin on endothelial function, including dose ranges and bioavailability considerations, are summarized in the table below.

Beyond its effects on vascular homeostasis, curcumin also modulates endothelial-dependent angiogenic pathways. Curcumin modulates angiogenic processes by regulating the expression of vascular endothelial growth factor (VEGF), thereby influencing endothelial cell proliferation and migration [11]. Importantly, the effects of curcumin on angiogenesis appear to be highly context-dependent. While it suppresses pathological angiogenesis in oncological settings primarily through the inhibition of VEGF signaling, under conditions of endothelial injury or ischemic stress, curcumin may contribute to the restoration of endothelial function by modulating redox balance and inflammatory pathways rather than directly promoting angiogenic formation. Importantly, in non-oncological conditions, curcumin does not appear to directly stimulate physiological angiogenesis; rather, its effects on endothelial repair and vascular remodeling are predominantly mediated through normalization of endothelial redox balance and inflammatory signaling, leading to the restoration of endothelial phenotype and function. This effect is particularly relevant in oncology, where pathological angiogenesis supports tumor growth but may also influence vascular remodeling in other disease states. In non-oncological settings, the available evidence does not support a direct pro-angiogenic effect of curcumin; instead, its influence on vascular remodeling appears to be secondary to improvements in endothelial redox balance and inflammatory status. Moreover, curcumin has demonstrated protective effects on the endothelium during anticancer therapy. Experimental studies indicate that curcumin reduces cisplatin-induced endothelial apoptosis and preserves vascular barrier integrity, suggesting a potential role as an adjuvant therapy to mitigate vascular toxicity [12].

Metabolic disorders, including type 2 diabetes mellitus, are closely associated with endothelial oxidative stress and chronic inflammation. Preclinical and clinical data indicate that curcumin improves endothelial function in diabetic patients by reducing reactive oxygen species production and enhancing NO-dependent signaling pathways [13,14]. These effects may translate into improved microvascular function and a reduced risk of vascular complications. A summary of selected studies evaluating the effect of curcumin on endothelial function, including dose and bioavailability, is provided in Table 1.

Curcumin is generally well tolerated, even with long-term supplementation; however, its clinical utility is limited by low oral bioavailability and heterogeneity among newer forms (e.g., piperine-enhanced, micellar, phytosomal), which complicates direct comparison of outcomes across clinical studies. Overall, curcumin exerts multifaceted beneficial effects on the vascular endothelium through antioxidant and anti-inflammatory actions, enhancement of nitric oxide bioavailability, and modulation of angiogenesis. From a clinical perspective, curcumin may represent a promising adjunctive strategy for improving endothelial function in populations at increased cardiovascular risk, particularly in the context of aging and metabolic disorders, although its routine clinical application remains constrained by form variability, bioavailability, and a further need for well-designed randomized trials [10].

## 4. Lipid Metabolism

Curcumin, a polyphenolic compound derived from *Curcuma longa*, has been widely studied for its regulatory effects on lipid metabolism and its potential role in the prevention and treatment of metabolic disorders, including dyslipidemia, obesity, type 2 diabetes mellitus, and nonalcoholic fatty liver disease (NAFLD). Research published between 2020 and 2025 provides growing evidence that curcumin influences lipid homeostasis through both systemic and molecular mechanisms.

Early reviews published around 2020 highlighted curcumin’s capacity to modulate fatty acid synthesis, β-oxidation, and circulating lipid levels, primarily based on preclinical and small-scale clinical data [15]. Subsequent randomized controlled trials (RCTs) and meta-analyses confirmed these observations. By 2021–2022, systematic reviews suggested that curcumin supplementation was associated with reductions in total cholesterol (TC), triglycerides (TGs), and low-density lipoprotein cholesterol (LDL-C), with modest or inconsistent effects on high-density lipoprotein cholesterol (HDL-C), depending on dose and population characteristics [16]. More robust evidence emerged in 2023, when an umbrella review of RCTs demonstrated that curcumin significantly reduced TC, LDL-C, and TG while increasing HDL-C, particularly in individuals with metabolic disorders such as diabetes mellitus and metabolic syndrome. These effects were more pronounced when curcumin was administered for at least 8 weeks and in formulations with enhanced bioavailability [17]. Recent meta-analyses further confirmed curcumin’s lipid-lowering potential, reporting significant reductions in TGs, TC and body mass index (BMI), supporting its adjunctive role in managing dyslipidemia associated with metabolic disease [18].

At the cellular level, studies published in 2024 demonstrated that curcumin directly reduces lipid accumulation in hepatocytes. In vitro experiments using HepG2 cells showed that curcumin attenuated free-fatty-acid-induced steatosis through the regulation of the miR-22-3p/CRLS1 axis, highlighting a novel epigenetic mechanism linking curcumin to mitochondrial lipid metabolism [19]. In parallel, other studies indicated that curcumin suppresses hepatic de novo lipogenesis by downregulating sterol regulatory element-binding protein-1c (SREBP-1c), peroxisome proliferator-activated receptor gamma (PPARγ), and lipogenic enzymes such as acetyl-CoA carboxylase (ACC) and fatty acid synthase (FAS) [20]. The effects of curcumin on lipid metabolism can be broadly classified into direct mechanisms, including modulation of AMPK signaling and inhibition of SREBP-1c–mediated lipogenesis, and indirect mechanisms, such as attenuation of chronic inflammation and oxidative stress. Curcumin’s lipid-modulating effects are controlled by several interconnected signaling pathways. Activation of AMP-activated protein kinase (AMPK) has been consistently reported as a central mechanism, leading to increased fatty acid oxidation and inhibition of lipogenesis [20]. Additionally, curcumin influences nuclear receptors, such as PPARα and liver X receptor (LXR), contributing to improved lipid transport and cholesterol efflux [21]. Beyond direct metabolic regulation, curcumin’s antioxidant and anti-inflammatory properties reduce oxidative stress and chronic inflammation—key drivers of dyslipidemia and hepatic lipid accumulation [2].

Despite promising evidence, curcumin’s clinical application remains limited by its low oral bioavailability. Recent studies emphasize that nanoformulations, phospholipid complexes, and co-administration with piperine significantly enhance curcumin’s metabolic effects, including lipid-lowering effects [17,21]. Future large-scale, well-designed RCTs are required to establish standardized dosing regimens and to clarify long-term safety and efficacy. Overall, evidence from 2020 to 2025 indicates that curcumin exerts beneficial effects on lipid metabolism by improving circulating lipid profiles and modulating key molecular pathways involved in lipogenesis and fatty acid oxidation. However, its clinical efficacy appears to be strongly influenced by form, dosage, and treatment duration.

## 5. Inflammation

With ongoing demographic and epidemiological transitions, the prevalence of chronic inflammatory diseases is increasing. Those diseases are often either accompanied by an inflammation or their management is harder for patients when inflammation occurs, therefore generating higher social costs. Patients frequently seek help in dietary supplements and natural, herbal medicine. Curcumin is a great example of an anti-inflammatory, natural agent that may be found in many ready-to-use formulations. Some of the most popular chronic diseases, like diabetes mellitus (DM) or chronic kidney disease (CKD), are especially prone to homeostasis deregulation due to inflammation. Pivari et al. investigated the influence of curcumin on patients with CKD, and the outcome was very promising—in comparison to an age- and sex-matched healthy control group without chronic kidney disease, the team acknowledged lowering of plasma levels of IL-4 (immune response modulator), MCP-1 (pro-inflammatory monocyte chemoattractant protein 1) and even IFN-ɣ, which plays a relevant role in CKD onset and progression. Also, uremic toxin levels and lipid peroxidation were lowered after 6 months of curcumin supplementation. The authors also mentioned a potential use of curcumin in neuro-inflammation and COVID-19 management [22]. The use of curcumin in post-COVID-19 patients seems to be very interesting as society still suffers from the effects of the pandemic. It has been shown that a 4-week supplementation of curcumin in patients who experienced COVID-19 infection and were vaccinated subsequently led to a decrease in inflammatory markers, i.e., IL-6 and MCP-1 [23].

Many authors have proven and confirmed curcumin’s positive influence on DM management by decreasing pro-inflammatory molecules, including TNF-α, IL-1β, and IL-6, but also by increasing antioxidant enzyme (i.e., glutathione peroxidase, superoxide dismutase) activity. The influence resulted in improved objective glycemic control indexes like fasting blood glucose level and HbA1C [24,25,26].

The results of Bańkowski et al. and Grafeneder et al. on curcumin and its performance may be worth exploring and further investigating. In their double-blinded RCTs, the authors proved that in groups of healthy subjects, curcumin supplementation did not affect IL-6 and TNF-α levels. These findings suggest that curcumin may preferentially modulate inflammatory pathways under pathological conditions rather than in physiological homeostasis [27].

Tiekou et al. analyzed the influence of co-administering iron formulations and curcumin. An observation performed on over 150 healthy participants led them to conclude that this combination exerts a measurable anti-inflammatory effect, especially lowering IL-6 and TNF-α levels. These effects are probably caused by the antioxidative aspect of curcumin’s influence on humans. However, this effect was observed in the context of iron supplementation, which is known to increase oxidative and inflammatory burden [28].

In a study on serious, life-threatening states, Mirjalili et al. performed an interesting double-blinded RCT on intensive care unit patients with some severe traumas. Most of the patients had improved biochemical and hematological parameters (i.e., potassium, BIL-T, CRP, WBC and PLT). The team also reported an increase in GCS and a trend toward lower mortality compared to the placebo group [29].

In conclusion, most of the authors focus on the influence of IL-6 and TNF-α, as curcumin mainly affects those molecules’ levels. Some of the authors have proven no influence on other molecules like PGE2 or CRP in humans, while curcumin-treated mice show increased levels of anti-inflammatory molecules like IL-10 and TGF-β and increased levels of CD4+ and CD25+ regulatory T cells [30,31,32].

## 6. Sepsis

Sepsis, as a particular kind of dysregulated inflammation caused by a serious infection, is a life-threatening state, and managing it may still be a challenge. Therefore, searching for new possible ways of curing it is important, and its treatment may represent another potential field of curcumin application. Its antibacterial and antiviral action and interactions with immune cells, e.g., dendritic cells, macrophages, lymphocytes B, T and NK—all these aspects suggest potential curcumin utility in serious conditions like sepsis [33].

Vieira et al. performed a double-blinded RCT on septic ICU (intensive care unit) patients. During the trial, they administered curcumin for 7 days straight. The outcomes received are promising, as they noted a beneficial effect in the biochemical and hematological parameters of the curcumin-treated patient group regarding BIL, pH, Hb, Hct, MCH, MCHC, and platelet levels. Also, a CRP level decrease was obtained. The authors also noted a lower death rate in the curcumin group; however, it was not statistically significant. Aspects like fluid intake and output and body temperature were also reduced in the intervention group [34]. Based on preclinical models, several authors suggest that curcumin may influence the PI3K/AKT pathway, which is connected to sepsis pathophysiology, as well as decrease the activity of proteins that are crucial for the apoptosis process, i.e., Bad, Bcl-xL, Cyto-c, Apaf1, and cleaved Caspase-3/6/9. A protective influence on crucial septic failure organs, like the liver, lungs, kidneys and heart, was also noted by different authors. Reduced fibrosis and improved collagen synthesis in the liver and reduced inflammation, bronchoconstriction, mucous secretion, cell necrosis and fibrin exudation in the lungs are the main mentioned mechanisms of curcumin performance [33,35,36].

The available data on the potential therapeutic effects of curcumin in sepsis are still limited and largely based on preclinical studies, animal models, or small clinical trials. Although these findings suggest a possible beneficial effect of curcumin on modulating the inflammatory response and oxidative stress, their interpretation remains limited due to methodological heterogeneity, small study sizes, and a lack of standardization of the doses and formulations used. Therefore, more well-designed clinical trials are necessary, particularly randomized, double-blind studies. Randomized, double-blind studies could also provide more conclusive data regarding the optimal dosage, duration of administration, and potential interactions of curcumin with other components of standard therapy for patients with sepsis. The results obtained in this way would be crucial for a reliable assessment of the true clinical significance of this substance and for its possible inclusion in therapeutic strategies for the treatment of sepsis.

## 7. Neuroprotection

Current neuroprotective approaches have become a new direction in the treatment of stroke and many neurodegenerative diseases. Therefore, there is an urgent need to develop an effective agent with neuroprotective properties, and curcumin demonstrated such properties in many studies. Its actions focus on the ability to combat oxidative stress, inflammation and the aggregation of toxic proteins in the brain. Curcumin acts through numerous molecular mechanisms, including activation of the Nrf2 and AMPK protective pathways and stabilization of the blood–brain barrier, as confirmed by both in vitro and in vivo tests. Scientists emphasize that modern nanotechnology is the key to the full therapeutic use of this polyphenol in regenerative medicine [37,38,39,40,41,42,43,44,45,46,47,48,49,50,51,52,53,54,55,56,57,58]. Several studies have analyzed and confirmed protective effects on mitochondrial function. Curcumin and its nanoformulas (CN) play a key role in restoring mitochondrial function, particularly after ischemia–reperfusion injury (IRI). Regarding its influence on mitochondrial enzymes (SDH, MDH, LDH), curcumin administration prevents the decline in succinate dehydrogenase (SDH), malate dehydrogenase (MDH), and lactate dehydrogenase (LDH) levels. This protects the oxygenation chain and prevents mitochondrial damage. It also enhances the activity of mitochondrial complexes I, II, III, and IV [37,38,39,40,41,58,59,60,61,62]. Curcumin increases the level of mitochondrial proteins. UCP2 protein (uncoupling protein 2) helps regulate energy metabolism and reduce oxidative stress due to ischemia and maintains redox balance [37,39,43,48,50,55,56,59,61,62,63,64,65,66]. Curcumin stimulates antioxidant enzymes and GSH: this compound significantly increases the level and activity of enzymes such as superoxide dismutase (SOD), catalase (CAT), glutathione peroxidase (GPx), glutathione reductase, and glutathione S-transferase (GST) [39,41,43,44,47,48,49,50,51,52,53,54,58,63,66,67,68,69]. Other studies show that curcumin restores glutathione (GSH) levels, which is crucial for protecting neurons from cytotoxicity, but can also promote mitophagy (clearing of damaged mitochondria) by regulating the LC3-II/LC3-I protein ratio [39,43,44,61,63].

Curcumin’s action on oxidative stress is mediated by pro- and antioxidant enzymes. Curcumin acts as a powerful direct and indirect antioxidant. It increases the activity of superoxide dismutase (SOD), catalase (CAT), and glutathione peroxidase (GPx). It also restores reduced glutathione (GSH) levels, crucial for neuronal protection. It acts on the homoeoxygenase protein (HO-1) by activating the Nrf2 pathway. Curcumin and its derivative 27 induce overexpression of heme oxygenase-1 (HO-1) in the cytoplasm, which enhances cellular antioxidant defense. Curcumin directly neutralizes free radicals (ROS) and significantly inhibits nitric oxide (NO) production and the expression of inducible nitric oxide synthase (iNOS), limiting microvascular and neuronal damage [37,38,39,40,41,43,44,48,49,50,51,52,60,63,66,67,70].

Its neuroprotective effects include affecting inflammatory and signaling pathways, inhibiting inflammatory cascades by affecting a number of mediators and transcription factors. It is a potent inhibitor of NF-κB, which leads to a decrease in the production of pro-inflammatory cytokines such as TNF-α, IL-1β, IL-6, IL-8, and IFN-γ. Both curcumin and its derivative 27 significantly reduce nitric oxide (NO) production and the expression of inducible nitric oxide synthase (iNOS) [38,39,41,59,61,66,67,69,71,72,73,74].

Curcumin also inhibits cyclooxygenase-2 (COX-2) activity and prostaglandin E2 (PGE2) production, which reduces inflammation and neuronal damage. Curcumin modulates protein kinase pathways, increasing pERK/ERK, p38, and p-MEK expression ratios, promoting cell survival. It inhibits the JNK pathway, preventing the loss of dopaminergic neurons. By activating the PI3K/AKT pathway, curcumin regulates cell growth and death. By activating Nrf2, it induces the expression of heme oxygenase-1 (HO-1), enhancing antioxidant defense. Curcumin increases the expression of TFEB, a key regulator of the autophagy–lysosomal pathway (ALP), leading to increased lysosomal biogenesis and cell clearance. It activates the PI3K/AKT pathway, promoting cell survival and inducing the synthesis of BDNF, essential for neurogenesis and synaptic plasticity [37,38,39,40,41,43,47,48,50,56,57,58,62,66,69,70,75,76,77].

Curcumin promotes the proliferation and differentiation of neural stem cells (NSCs). In the hippocampal region, it restores impaired neurogenesis by regulating the Wnt/β-catenin pathway and increasing BDNF expression and CREB phosphorylation. It also reduces markers of astrocyte activation (GFAP) and microglia (Iba-1, TSPO), thereby resolving chronic inflammation. Curcumin also promotes microglial polarization from a pro-inflammatory M1 to a protective M2 phenotype. Its anti-apoptotic effect involves increasing Bcl-2 expression while decreasing Bax, p53, and caspase-3 levels. It modulates the response of macrophages and lymphocytes (T, B, Th17), protecting the blood–brain barrier from infiltration, and protects neurons in the hippocampal region, improving memory and cognitive function [40,43,48,50,51,52,67,69].

Curcumin is considered a potential treatment for SM, HD, PD, SLA, ALS, AE and AD due to its ability to inhibit the aggregation of β-amyloid peptide (Aβ) and destabilize existing amyloid plaques. It lowers the levels of the BACE1 enzyme (key in amyloid production) and inhibits the hyperphosphorylation of tau protein [38,40,41,47,48,49,50,51,52,53,56,57,58,60,62,66,68,69,70,71,78,79]. As mentioned above, curcumin stimulates neurogenesis by promoting the proliferation and differentiation of neural stem cells (NSCs) through activation of the Wnt/β-catenin pathway and increased expression of BDNF and CREB. It reduces microgliosis and astrogliosis, thus calming chronic inflammation in the brain. In PD models, curcumin protects dopaminergic neurons from degeneration induced by neurotoxins (e.g., MPTP, 6-OHDA, rotenone) and also inhibits the aggregation of alpha-synuclein, which prevents the formation of Lewy bodies. It induces autophagy by inhibiting the AKT/mTOR pathway (which allows for the removal of damaged organelles and proteins). As a result, it improves motor performance (coordination and movement speed) in animals. In human studies focusing on nanomicelles, a trend toward improved motor function has been observed. Curcumin has a strong immunoregulatory effect, which is crucial in MS and its animal model (EAE)—it protects the integrity of the BBB, limiting the penetration of autoreactive Th17 lymphocytes into the central nervous system. It blocks IL-12 production and signaling (JAK-STAT pathway), which inhibits the Th1-dependent inflammatory cascade. Ultimately, it supports myelin sheath repair by stimulating oligodendrocyte differentiation. Curcumin mitigates the effects of ischemia–reperfusion injury (IRI) by reducing the area of infarction and cerebral edema. It restores the activity of enzymes such as SOD, CAT, and glutathione (GSH) levels by neutralizing free radicals generated during reperfusion. It protects the structure and function of mitochondria (SDH, LDH, and MDH enzymes), preventing neuronal energy deficits. In amyotrophic lateral sclerosis (ALS), curcumin has a protective effect on astrocytes and motor neurons, which degenerate in this disease. Curcumin also acts as a chelator, binding heavy metal ions and reducing their toxicity, for example, by attenuating mitochondrial dysfunction and reducing oxidative stress induced by aluminum chloride. It protects the hippocampus from damage caused by lead, cadmium, and mercury. It alleviates myelin damage induced by bisphenol A (BPA). It shortens the duration of seizures, reduces inflammatory cytokine levels (TNF-α) in the hippocampus, and increases dendritic spine density and reduces memory deficits in Huntington’s disease. In diabetic neuropathy, it promotes nerve fiber regeneration by increasing the expression of nerve growth factor (NGF) [37,38,39,40,41,43,47,56,59,62,66,67,68,69,71,72,73,74,75,79,80,81,82,83,84,85,86,87,88,89,90,91,92].

The main obstacle to the therapeutic use of curcumin is its low bioavailability, absorption and solubility. Curcumin is hydrophobic and nearly insoluble in water at physiological pH. Rapid metabolism and elimination after oral administration result in fast glucuronidation and sulfation in the liver and intestine. Although curcumin is lipophilic, the concentration that reaches brain tissue after standard administration is usually very low (nanomolar) [38,39,43,48,50,62,66,67,69,90,92].

To enhance the neuroprotective effects of curcumin and overcome its limitations, derivatives such as ferritin nanocages (HFnCUR), nanoformulations, nanoemulsions, nanoparticles, nanomicelles and analogues have been developed. Derivative 27, synthesized to improve efficacy at low doses, demonstrates better permeability across the blood–brain barrier (BBB) than natural curcumin and effectively reduces Aβ levels and inflammatory markers in AD models. Tetrahydrocurcumin (THC) is an active metabolite of curcumin (promoting the growth of beneficial bacteria, such as Bifidobacteria and Lactobacilli), which exhibits potent antioxidant activity, prevents apoptosis, and supports recovery from brain injury. Bisdemethoxycurcumin (BDMC) exhibits potent anti-inflammatory and anti-amyloidogenic properties, surpassing the activity of curcumin itself in some in vitro tests. Several studies are in progress to develop advanced delivery systems, such as nanotechnology using polymer nanoparticles, liposomes, micelles, and ferritin nanocages (HFn-CUR), which allow for increased stability, better solubility, and more effective BBB crossing. Adjuvants have also been used, i.e., the simultaneous administration of curcumin with piperine (a component of pepper), which inhibits the enzymes responsible for its metabolism and can increase curcumin’s bioavailability in humans by up to 2000% [38,40,41,43,48,50,56,57,58,60,61,62,64,65,71,72,80,82,89,92]. The results of tests on cells and tissues are presented in bold.

Curcumin, a natural, inexpensive product with proven pharmacological safety, has been shown to have neuroprotective effects on the brain. Due to its influence on epigenetic processes, it can be considered an appropriate and accessible treatment option for many neurodegenerative diseases. Studies focusing on the effects of curcumin are presented in Table 2.

## 8. Limitations

The results of studies assessing the efficacy of curcumin remain equivocal, due to significant methodological differences between individual studies. This heterogeneity encompasses the characteristics of the study populations, the formulation of the compound used, the duration of exposure, the choice of endpoints, and the level of risk of bias. Despite promising scientific evidence supporting the health benefits of curcumin, its clinical use remains limited due to its low oral bioavailability and lack of standardized doses. Recent studies highlight that nanoformulations, phospholipid complexes, and co-administration with piperine significantly enhance curcumin’s metabolic effects, including lipid-lowering effects [3,9]. The clinical efficacy of curcumin appears to be strongly dependent on the formulation, dosage, and duration of treatment. The possibility of interactions with other drugs and dietary supplements should also be considered. Further large-scale, well-designed RCTs are needed to establish standardized dosing regimens and clarify long-term safety and efficacy.

One significant limitation to the potential neuroprotective effects of curcumin is its relatively low bioavailability and limited ability to cross the blood–brain barrier. This barrier is a highly selective physiological structure that limits the transport of many compounds into the central nervous system. Curcumin has been shown to have limited penetration across the blood–brain barrier, which may result in insufficient concentrations in brain tissue. Consequently, this phenomenon may constitute a significant limitation in research on the neuroprotective effects of curcumin, particularly in the context of clinical applications.

## 9. Conclusions

Curcumin has a beneficial effect on the function of the vascular endothelium, primarily by improving its elasticity and reducing oxidative stress, which may contribute to a reduced risk of developing cardiovascular disease. Studies suggest that regular curcumin supplementation may support endothelial integrity, preventing damage caused by external factors such as inflammation and excessive production of reactive oxygen species. Numerous studies have shown that curcumin can significantly reduce total cholesterol, LDL-C, and triglyceride levels while increasing HDL-C levels, particularly in individuals with metabolic disorders such as diabetes and metabolic syndrome. Improved lipid metabolism may lead to a reduced risk of atherosclerosis and other dyslipidemia-related cardiovascular diseases. Curcumin’s potential antibacterial and antiviral effects, as well as its interactions with immune cells such as dendritic cells, macrophages, B, T, and NK lymphocytes, offer potential use in the treatment of severe infections such as sepsis. Curcumin likely exerts neuroprotective effects through multiple molecular mechanisms, including activation of the Nrf2 and AMPK protective pathways and stabilization of the blood–brain barrier, as supported by in vitro and in vivo studies. Curcumin may potentially contribute to improved cardiovascular health and reduced risk of diseases related to endothelial dysfunction, inflammation, lipid abnormalities, infections, and neurological diseases. Although many results from previous studies are promising, further clinical trials are necessary to fully define the optimal dosage of curcumin and the precise indications for its use in specific clinical situations.

## Figures and Tables

**Table 1:** Summary of selected studies assessing the effects of curcumin on endothelial function with dose and bioavailability considerations.

Author/Year	Study Population/Model	Dose and Duration	Endothelial Assessment	Main Findings	Formulation/Bioavailability Considerations
Santos-Parker et al., 2017 [6]	Healthy middle-aged and older adults (45–74 years)	150 mg/day, 12 weeks	FBFACh, brachial artery FMD	Improved endothelial function; increased NO bioavailability; reduced oxidative stress	Bioavailable curcumin extract; not directly comparable to standard curcumin powder
Santos-Parker et al., 2018 [7]	Healthy middle-aged and older adults without CVD	150 mg/day, 12 weeks	FMD, response to L-NMMA and vitamin C	Improved NO-dependent vasodilation; reduced oxidative suppression	Enhanced bioavailability formulation; optimized pharmacokinetics
Rungseesantivanon et al., 2010 [8]	Diabetic animal model (rats)	~100–300 mg/kg/day	Endothelial markers, oxidative stress	Improved endothelial function; reduced superoxide production	Supraphysiological doses; not directly translatable to humans
Mad Azli et al., 2024 [13]	Patients with cardiovascular risk factors (meta-analysis)	~80–1500 mg/day, 4–24 weeks	Indirect vascular and metabolic markers	Improved lipid and vascular parameters	Highly heterogeneous doses and formulations; limited dose–response inference
Kobutree et al., 2019 [12]	Endothelial cells exposed to cisplatin (in vitro)	1–10 µM	Vascular barrier integrity, cytotoxicity	Protection against cisplatin-induced endothelial damage	Concentrations exceed achievable plasma levels; mechanistic evidence only

**Table 2:** Studies focusing on the effects of curcumin.

Type of Study	Study Material	Curcumin Dose	Study Duration	Conclusions
38. In vivo	48 Wistar rats	25 mg/kg m c	5 days orally	Reduction in reactive oxygen species (ROS) and increased activity of antioxidants and mitochondrial enzymes. This, in turn, increased the pERK/ERK expression ratio and TEFB expression. IRI restored the activities of the antioxidant enzymes CAT and superoxide dismutase to the levels of healthy control rats, indicating a significant enhancement in antioxidant capacity and increased GSH content after curcumin administration.
39. In vivo 39. In vivo	Mice APP/PS1, *n* = 28	Derivate 27, (50 mg/kg/day), orally in the form of a gelatinous carrier Mouse microglial cell line	28 days 18 h	Improved short-term spatial memory and significantly reduced the levels of Pro-IL-1β and amyloid precursor protein in the hippocampus, as well as the levels of Aβ in the hippocampus and plasma; more permeable through the BBB than curcumin. Derivative 27 significantly reduced NO production and the levels of pro-inflammatory proteins, inducible NO synthase, pro-interleukin-1β (Pro-IL-1β) and cyclooxygenase-2. It also activated nuclear factor erythroid 2-related transcription factor 2 (Nrf2) and significantly increased the levels of Nrf2 and heme oxygenase-1 protein in the nucleus and cytoplasm, respectively. It was more permeable through the BBB than curcumin alone.
78. In vivo	Male rats	30 mg/kg of curcumin, intraperitoneally	8 weeks	Reduced oxidative stress in order to decelerate the aging process. Decreased brain lipid peroxidation and increased SOD.
95. In vivo	Male rats	200 mg/kg b.w., oral	6 weeks	Cur significantly enhanced the level of antioxidants and considerably lowered the level of oxidative stress markers. It also increased the activity of electron transport chain complexes in the mitochondria of aged brain tissue, demonstrating its antioxidant potential at the mitochondrial level
96. In vivo	Mice	20–160 mg/kg	30 days	Reduced anxiety, inflammation, and oxidative damage in the prefrontal cortex; increased viable neurons in cadmium-induced neurotoxicity.
96. In vivo	Male rats	25 lub 50 mg/kg	21 days	Protected against motor deficits and neurochemical changes in neurotoxicity; piperine significantly enhanced the effect of curcumin.
97. In vitro	Cell lines in molecular models of AD	15–30 µM 5–15 µM	In short trials (<24 h), periods (4–6 days)	Limited the formation of beta-amyloid (Aβ) fibrils and reduced oxidative damage.
97. In vitro	Cell lines in molecular models of PD	15–30 µM 5–15 µM	In short trials (<24 h), periods (4–6 days)	Protection cerebral astrocytes from LPS-induced toxicity by inhibiting the expression and activity of CYP2E1, which reduced reactive oxygen species (ROS) levels. Protection of PC12 cells from apoptosis induced by MPP+ and MPTP. Efficacy was demonstrated at low concentrations.
97. In vivo	PD mice	150 mg/kg body weight daily, orally	1 week	Reversal of GFAP and iNOS protein expression, decrease in pro-inflammatory cytokine levels in the striatum and improvement in motor performance.
96. Clinical trials	People, healthy individuals *n* = 40	Theracurmin (90 mg 2×/daily)	18 months	Improved memory and attention; less amyloid accumulation in the amygdala and hypothalamus.
96. Clinical trials	AD patients *n* = 36	2 or 4 g/day	24 weeks	No significant differences compared to a placebo, which the authors attributed to the low bioavailability of the standard form.
96. Clinical trials	AD patients *n* = 48	Curcumin–galactomannan complex (400 mg 2×/day	6 months	Improved MMSE and GLFS test results; favorable change in biomarker levels (BDNF, Aβ42, tau).
96. Clinical trials	PD patients *n* = 60	Nanomicelle (80 mg/day	9 months	No significant clinical differences between the study group and placebo.
42. In vitro	In HEK293 cells with the Swedish mutation (APPSw)	5 µM	24 h	Reduced Aβ42 production by 35.7%.
42. In vivo	Mice Tg2576 (model AD)	0.016% curcumin in the diet 0.05% curcumin in the diet	6 months	Reduced the insoluble and soluble Aβ and amyloid plaque burden; significantly reduced plaques in the cortex and hippocampus.
42. In vivo	Mice APPSw/PS1dE9	7.5 mg/kg body weight (bw) administered intravenously	7 days	Cleansed and reduced existing plaques and reversed structural changes in dendrites.
42. In vivo	Sprague Dawley rats (infuzja Aβ):	0.05% in the diet Intrahippocampal injection, dose: 30 mg/kg body weight	5 months 5 days	Improved spatial memory deficits and reduced plaque burden. Increased PSD95 mRNA levels by 218% and improved performance on the Morris water maze test.
42. In vivo	Wistar Rats	30 i 60 mg/kg orally	6 weeks	Decrease in MDA and NO levels in the brain and increase in the activity of antioxidant enzymes (SOD, catalase, GPx).
84. in vivo	Mice APPSw/PS1dE9	7,5 mg/kg (BW) to the tail vein	7 days	Removal of or reduction in existing atherosclerotic plaque burden in the cerebral cortex, as well as significant reversal of amyloid-related dystrophic structural changes in neuronal dendrites.
88. In vitro	Human embryonic kidney cells transfected with Swedish mutant APP (APPSw)	Cur 5 µM	24 h	Significantly reduced Aβ42 production in APPSw HEK293 cells by 35.7%. Curcumin did not significantly affect APP mRNA, BACE1 mRNA, or BACE1 protein levels.
89. In vivo	Wistar rats	400 mg/kg	10 days	Protected against quinolinic acid-induced neurodegeneration by restoring intranuclear Nrf2 levels as well as total SOD and GPx activity in the brain. As a result of Nrf2 induction, curcumin-treated rats had lower protein carbonyl levels in the striatum, indicating less oxidative stress in these rats compared to the control group.
71. In vivo	Male Sprague Dawley rats, *n* = 60	100 mg/kg and Ic, 300 mg/kg, 30 min before surgery and daily by intraperitoneal injection		Curcumin alleviated symptoms of nerve damage and infarct volume, reduced water content in the brain, attenuated neuronal apoptosis, and also increased the expression of p-MEK, p-ERK, p-cREB, Bcl-2 and decreased the level of Bax, within 24 h of ischemia–reperfusion, especially at higher doses.
64. In vitro	PC12 cells with ischemia	Cur (0, 1,25, 5,0, 20 μmol/l)	24 exposure hours	Neuroprotective effect of curcumin, associated with the increase in UCP2 protein level and inhibition of oxidative stress induced by chronic cerebral ischemia.
45. In vitro	bEnd.3 i HT22 cells	5 μM, 10 μM, 20 μM i 40 μM	24 exposure hours	Lower doses significantly increased the viability of bEnd.3 cells treated with OGD/R. LDH assays showed that curcumin significantly inhibited LDH release. Curcumin inhibited the inflammatory response and cell apoptosis induced by OGD/R. No significant changes in IL-6, TNF-α, and IL-1β levels were observed after curcumin treatment in bEnd.3 and HT22 cells. Dose of 40 µM significantly decreased cell viability.
51. In vitro	HEK293-Tau3R and SH-SY5Y cell viability study	Cur and piperlongumine hybrids Reactive oxygen species (ROS) scavenging assays: concentrations ranging from 0.1 to 10 µM were used Nrf2 induction: concentrations of 0.3, 3, 10, and 30 µM were tested		The compounds exhibited good scavenging properties, inhibiting the aggregation of the Tau peptide PHF6 and demonstrating activity against LPS-induced inflammation. These compounds exerted neuroprotective effects against oxidative stress induced by rotenone–oligomycin treatment, as well as against okadaic acid-induced Tau hyperphosphorylation.
54. In vitro 54. In vivo	Primary rat brain microvascular endothelial cells (RBMECs) and rat cerebral cortex astrocytes (RCAs) Mice	Cur enclosed in ferritin nanocages, HFn solution (1 mg/mL, NaCl 150 mM 1 mg/kg HFn-CUR in the form of intraperitoneal injection at a dose of 0.1 mg/ml		Mild beneficial effects on cognitive performance. Furthermore, it effectively reduced microgliosis and astrogliosis in vivo in mice, suggesting potential neuroprotective benefits. Safe and effective in reducing inflammation. It enhanced cellular responses to inflammation, reduced RAGE-dependent stress and had a mild beneficial effect on cognitive performance. It did not directly affect amyloid plaques and effectively reduced microgliosis and astrogliosis.
74. In vitro 74. In vivo	Telencephalon tissue isolated from mouse embryos APP/PS1 double-transgenic mice *n* = 30	Medium with different concentrations of curcumin (0 µM, 0.5 µM, 2.5 µM, 12.5 µM and 62.5 µM 100 and 300 mg/kg), intraperitoneal	12 days 7 days	At a concentration of 0.5 µM, the highest proliferative capacity of cells in neurospheres was observed. The 2.5 µM group produced the largest number of neurospheres, suggesting a potential increase in the cloning capacity of neural stem cells. At a concentration of 62.5 µM, a clear cytotoxic effect was observed. Significant inhibition of neurosphere proliferation. Improved cognitive dysfunction—escape latency time in AD mice in the 100 mg/kg group was significantly shorter. Significantly increased neuronal regeneration in the hippocampal region of mice.
79. In vivo	Wistar rats, *n* = 38	40/80/160 mg/kg/d intragastric	2 weeks	Medium and high doses attenuated abnormal movements in the APO-induced rotation test and prolonged latencies—high-dose curcumin treatment could protect dopaminergic (TH-positive) neurons from damage by 6-OHDA, which provided histological evidence.
90. In vivo	PD rats, rotenone-induced	Intraperitoneally at a dose of 200 mg/kg/day	3 weeks	Significant reduction in neuronal activity. Cur can improve motor impairments and electrophysiological parameters and may be beneficial in the treatment of PD.
91. Clinical trials	Patients	Cur molecule, TML-6 oral dose of 150 mg/kg	4 months	Improved the stability and metabolism of curcumin. Cell biological studies demonstrated that TML-6 could inhibit the synthesis of the β-amyloid precursor protein and β-amyloid (Aβ), upregulate Apo E, suppress NF-κB and mTOR, and increase the activity of the antioxidative Nrf2 gene, resulting in significant improvement in learning, suppression of the microglial activation marker Iba-1, and reduction in Aβ in the brain.
74. In vitro 74. In vivo	Isolated telencephalon tissue from mouse embryos, *n* = 16 Mice, *n* = 30	0.5, 2.5, 12.5, 62.5 µM intraperitoneally 100, 300 mg/kg	48 h 7 days	At a concentration of 0.5 µM, it showed the highest proliferative capacity of cells in neurospheres; at a concentration of 2.5 µM it produced the largest number of neurospheres, which suggests a potential increase in the ability to clone neural stem cells; at a concentration of 62.5 µM, there was a clear cytotoxic effect, showing significant inhibition of neurosphere proliferation. Cur reduced Aβ accumulation in the hippocampus and cerebral cortex. Molecular studies showed that curcumin increased BDNF expression and enhanced CREB phosphorylation.
82. In vivo	Wild mice—A model of chronic neuroinflammation, *n* = 91	35,70 i 140 mg/kg fitosomal Cur		Reduction in neuro-inflammatory markers—better bioavailability. The highest dose was most effective in reducing markers of neuroinflammation.
66. In vivo	Male mice C57BL/6J—Alzheimer’s disease model induced by injection of beta-amyloid Aβ1-42	100 mg/kg/day	21 days	Significantly alleviated Alzheimer’s disease symptoms and improved neurological function: improved learning and memory performance in the Morris water maze test. Cur restored impaired neurogenesis in the dentate gyrus (DG) of the hippocampus. An increased number of new neurons was observed.
66. In vivo	Mice ICR (Parkinson’s D.) Mice SJL/J (SM)	200 mg/kg i.v. Intravenous injections of 50 or 100 µg	7 days 25 days	Increase in SOD1 expression, inhibition of glial response and reduction in astrocyte activation. Alleviated the severity of clinical paralysis and reduced demyelination processes in the CNS.
98. In vivo	Episodic migraine patients, *n* = 74	Nanocurcumin, in combination with ω-3 fatty acids	2 months	ω-3 fatty acids and nanocurcumin can reinforce each other’s effects in the downregulation of COX-2/iNOS mRNA, as well as reduce their serum levels.
86. Clinical trials	Double-blind, randomized, placebo-controlled trial ALS	Nanocurcumin p.o. 80 mg/day	12 months	Improved the survival curve. In ALS, the results suggested that nanocurcumin is safe and might improve the probability of survival as an add-on treatment in patients with ALS, especially in those with existing bulbar symptoms.
65. Clinical trials	Patients with neurodegenerative diseases, *n* = 25	500–8000 mg daily	3 months	Improved cognitive function, regulated neurotransmitter levels (dopamine, serotonin) and helped remove amyloid-beta plaques by stimulating phagocytosis. A dose of up to 8 g per day is considered safe.
69. In vivo	Wistar rats with SH-SY5Y neuroblastoma cells (AD model) *n* = 40	80 mg/kg orally	3 weeks	Cur reduced Aβ generation and α-synuclein-induced cytotoxicity.
50. In vivo	Rat model 6-OHDA	40, 80 lub 160 mg/kg	14 days	A dose of 160 mg/kg significantly improved motor function and protected dopaminergic neurons.
53. In vivo	*n* = 30, rats (Wistar, Sprague Dawley, Lewis)	80 mg/kg (p.o.)	3 weeks	Anti-inflammatory effect, neutralized free radicals. Antioxidant properties: Curcumin activated the Nrf2 pathway, which increased the production of defensive enzymes such as catalase, superoxide dismutase (SOD), and glutathione peroxidase.
87. Clinical trials 89. Clinical trials	Older adults without dementia (51–84 years) *n* = 40 Patients with cognitive decline or possible AD (over 50 years)	90 mg of Cur in the form of Theracurmin, orally 1 g or 4 g of Cur in powder or capsule form	18 months 6 months	Improvements in memory and attentional performance were observed. Protection against neuropathological deposits in the amygdala and hypothalamus. Focus on bioavailability, demonstrating higher effects when in capsules than in powder form.
94. In vivo 83. In vivo 92. In vivo 93. In vivo 94. In vivo	3x-Tg-AD mice Alzheimer’s disease Male albino rats Parkinson’s disease Female C57BL/6 mice Multiple sclerosis Male Sprague Dawley rats Spinal cord injury Female Wistar Hannover rat stroke	150 mg/kg 30 mg/kg 20 mg/kg 100 mg/kg 300 mg/kg		Improved behavior, inflammation, and Aβ accumulation in a mouse model of AD. Decreased neurotoxic effects, degenerative histological changes, and OS in a PD rat model’s cerebellar cortex. Decreased pro-inflammatory cytokine levels, and enhanced anti-inflammatory cytokine expression. Improved outcomes by decreasing the TLR4/NF-κB inflammatory signaling pathway in the SCI. Reduced neurological scores and apoptotic index in the ischemic group compared to the Cur-treated group.

## Data Availability

The data are available at the Department of Pharmacology and Therapeutics, Faculty of Medicine, Collegium Medicum in Bydgoszcz, Nicolaus Copernicus University, M. Curie 9, 85-090 Bydgoszcz, Poland.

## Abbreviations

Aβ	amyloid-β
ACC	acetyl-CoA carboxylase
AD	Alzheimer’s disease
AE	Autoimmune Encephalomyelitis
ALS	amyotrophic lateral sclerosis
AMPK	AMP-activated protein kinase
BACE1	β-site APP cleaving enzyme 1
BBB	blood–brain barrier
BDNF	Brain-Derived Neurotrophic Factor
BIL-T	total bilirubin
BMI	body mass index
CAT	catalase
CKD	chronic kidney disease
CV	cardiovascular
CVS	cardiovascular system
CVDs	cardiovascular diseases
COX-2	cyclooxygenase-2
CRP	C-reactive protein
Cur	curcumin
DM	diabetes mellitus
FAS	fatty acid synthase
FBFACh	Forearm Blood Flow response to Acetylcholine
FMD	Flow-Mediated Dilation
GCS	Glasgow Coma Scale
GPx	glutathione peroxidase
GSH	glutathione
GST	glutathione S-transferase
HbA1C	glycated hemoglobin
HD	Huntington’s disease
HDL-C	high-density lipoprotein cholesterol
HO-1	homoeoxygenase protein
ICU	intensive care unit
IL-1β	interleukin-1β
iNOS	nitric oxide synthase
LDL-C	low-density lipoprotein cholesterol
L-NMMA	NG-monomethyl-L-arginine
LXR	liver X receptor
INOS	inducible NO synthase
MCP-1	pro-inflammatory monocyte chemoattractant protein 1
NAFLD	nonalcoholic fatty liver disease
NF-κB	nuclear factor kappa-light-chain
NGF	nerve growth factor
Nrf2	nuclear factor erythroid-derived 2-like 2
NO	nitric oxide
Nrf2	nuclear factor erythroid 2-related factor 2
PD	Parkinson’s disease
PLT	platelet
PPARγ	peroxisome proliferator-activated receptor gamma
RCTs	randomized controlled trials
SREBP-1c	sterol regulatory element-binding protein-1c
SLA	amyotrophic lateral sclerosis
SOD	dismutase
SM	Sclerosis Multiplex
TC	total cholesterol
TFEB	Transcription Factor EB
TG	triglyceride
TNF-α	tumor necrosis factor-α
UCP2 protein	mitochondrial uncoupling protein 2
VEGF	vascular endothelial growth factor
WBC	white blood cell
